# Macro- and Micro-Analysis of Factors Influencing the Performance of Sustained-Release Foamed Cement Materials

**DOI:** 10.3390/ma18143330

**Published:** 2025-07-15

**Authors:** Yijun Chen, Shengyu Wang, Yu Zhao, Pan Guo, Lei Zhang, Yingchun Cai, Jiandong Wei, Heng Liu

**Affiliations:** 1 Henan Central Construction Engineering Co., Ltd., Zhengzhou 450047, China; 2 School of Water Conservancy and Transportation, Zhengzhou University, Zhengzhou 450001, China

**Keywords:** sustained-release foamed cement, performance study, admixtures, durability

## Abstract

This paper addresses the issues of insufficient expansion force, low early strength (1-day compressive strength < 1.5 MPa), and poor toughness (flexural strength < 0.8 MPa) in traditional chemical foamed cement used for road grouting repair. By combining single-factor gradient experiments with microscopic mechanism analysis, the study systematically investigates the performance modulation mechanisms of controlled-release foamed cement using additives such as heavy calcium powder (0–20%), calcium chloride (0.2–1.2%), latex powder (0.2–1.2%), and polypropylene fiber (0.2–0.8%). The study innovatively employs a titanium silicate coupling agent coating technique (with the coating agent amounting to 25% of the catalyst’s mass) to delay foaming by 40 s. Scanning electron microscopy (SEM) and pore structure analysis reveal the microscopic essence of material performance optimization.

## 1. Introduction

With the rapid development of road traffic infrastructure, China has gradually emerged as one of the most advanced transportation powerhouses in the world. By the end of 2023, the total length of highways and expressways in China had reached 5.4368 million kilometers, ranking first globally in both categories [1]. With rapid economic growth, traffic volume continues to increase. Consequently, pavement distress caused by heavy loads and environmental factors has become increasingly severe, posing greater challenges for highway maintenance. In 2022, 5.3503 million kilometers of highways were maintained, accounting for 99.9% of the total national highway mileage [2]. Throughout the service life of roads, common types of pavement distress include cracks, voids, subsidence, ruts, and bridgehead bumping, which significantly affect driving quality, safety, and comfort [3]. Subgrade distress typically results from several factors, including insufficient subgrade bearing capacity, excessive loads from heavy vehicles, and harsh environmental conditions. Among the various types of pavement distress, distress caused by poor subgrade bearing capacity is particularly common. In engineering practice, two main technical approaches are often used to address these issues: excavation repair and non-excavation repair [4,5]. Owing to its simplicity, low construction requirements, and rapid traffic restoration, the non-excavation grouting method has become the main technique for subgrade reinforcement and maintenance [6].

Foamed cement grouting is a modern technique for non-excavation road restoration. This method involves introducing gas into a cement slurry to form a lightweight foamed material. Once cured, this material effectively absorbs and disperses impact loads, making it widely used for construction and subgrade reinforcement across various European and Asian countries [7,8]. However, chemical foamed cement gradually generates foams during mixing, leading to insufficient expansion force during grouting. Consequently, the foamed cement cannot fully expand and compact to fill voids in the road base. Regarding mechanical properties, chemical foamed cement typically exhibits lower strength and poorer toughness than physical foamed cement, making it prone to brittle fracture and potential secondary damage. To address these limitations, several researchers have explored the use of physical foamed cement for road grouting [9]. However, the poor fluidity of this cement prevents the slurry from filling voids in the road base, making it unsuitable for effective road repair. Chemical foaming allows the rapid injection of the slurry into the road base and subsequent foaming after grouting. This process enables the material to fully utilize its expansion force, thereby enhancing its filling and compaction performance. Therefore, chemical foaming has received widespread attention [10,11,12].

Foamed cement is a lightweight, porous material formed through the incorporation of tiny bubbles into a cement slurry via chemical or physical techniques. This process involves mixing foaming agents and stabilizers into the slurry, stirring to evenly distribute the bubbles, followed by pouring and curing [13,14,15]. Foamed cement mainly comprises binding materials, foaming agents, stabilizers, and admixtures, which are critical for achieving the desired material density. Foamed cement, a lightweight and multifunctional material, has exhibited excellent performance in practical applications, particularly in thermal insulation, soundproofing, and lightweight filling [16,17]. However, in road grouting projects, limited research has addressed specific performance requirements such as self-expansion capability, early strength, flowability, and toughness. To address these challenges, strategies such as optimizing the matrix material ratios and incorporating functional admixtures have been used to improve foaming uniformity, rheological properties, mechanical strength, and durability. These improvements significantly enhance material suitability for complex engineering applications [18,19,20,21].

Sustained-release technology was first developed to improve drug delivery. Through specific technical methods, active compounds are encapsulated within sustained-release materials. In a reaction system, these materials gradually dissolve or become permeable, enabling the release of the encapsulated compound at a controlled rate. This delayed release enhances the efficacy of the active compound and reduces side effects. Currently, sustained-release technology is widely applied in agriculture, food processing, coatings, and water treatment [22,23,24,25]. In the cement and concrete industry, this technology is mainly used to regulate properties such as curing, crack self-healing, and fluidity retention [26]. Titanate coupling agents, which evolved from silane coupling agents, are widely used in coatings, rubber, adhesives, and related fields [27,28,29]. Owing to their ability to gradually shed hydrophobic layers through the hydrolysis of chemical bonds in water, these coupling agents are suitable for use in sustained-release foamed cement. Upon application to foaming agents or catalysts, titanate forms a hydrophobic coating, which hinders their dissolution in cement paste, enabling controlled and sustained foaming. Overall, different grouting materials provide specific advantages for various applications but have certain limitations. Foamed cement, a lightweight and cost-effective material, exhibits promising potential for road grouting repair. Controlled-release technology has significantly advanced in concrete applications, thereby improving strength, durability, and workability. However, the application of this technology in foamed cement remains at an early exploratory stage. Therefore, the incorporation of controlled-release technology into foamed cement systems is a promising approach. Encapsulating and modifying foaming components to achieve controllable bubble generation and the use of functional admixtures can enhance the physical and mechanical properties of the material. This study provides a new strategy for developing adjustable, lightweight, and high-strength multifunctional foamed cement. Based on the above research background and technical challenges, this study aims to develop a sustained-release foaming cement material suitable for road grouting repair. Through systematic study of the regulation mechanism of admixtures such as heavy calcium powder, calcium chloride, latex powder, and PP fiber on the performance of the material, the following specific objectives are achieved: Establishing the slow release foaming technology system to solve the problem of insufficient expansion force of traditional chemical foaming cement. Optimizing the material ratio to meet the engineering requirements of early strength (1 d compressive strength ≥ 2 MPa) and flow (≥180 mm) for road grouting repair. Through microstructure regulation, the durability (water absorption rate ≤ 6%) and crack resistance (90 d flexural strength ≥ 1 MPa) of the material are improved. It provides theoretical support and technical solutions for the application of foam cement in road engineering scenes such as subgrade reinforcement and bridge head bump treatment.

Currently, different types of grouting materials each have their own advantages in various scenarios, but they also come with certain limitations. Foam cement, due to its lightweight and low cost, has shown potential in road grouting repair. The controlled-release technology has achieved breakthroughs in the concrete industry by enhancing strength, durability, and construction performance. This paper, based on the construction needs of grouting materials, extends the controlled-release technology to the foam cement system. Through single-factor experiments, the study focuses on adjusting the properties of cementitious materials, treating the foam components, and optimizing the performance of composite admixtures. This resulted in the development of a cement-based grouting material that can release foam, has stable strength, low water absorption, and good toughness.

## 2. Materials and Methods

### 2.1. Experimental Materials

Various raw materials were used to prepare the sustained-release foamed cement. CA50-iii (A700) aluminum acid cement (Jia Nai Special Alkali Company, Zhengzhou, Henan Province, China) was used as the cementitious material. The material had a specific surface area of 397 m^2^/kg. The main chemical composition and performance indicators are shown in Table 1 and Table 2.

### 2.2. Preparation of Deflating Foamed Cement

To achieve controlled-release foaming, manganese dioxide was first coated with a titanium ester coupling agent. Subsequently, dry powders (such as gelling materials, foaming agents, stabilizers, and catalysts) were mixed in a container according to a specified ratio. Water at the appropriate temperature was then added, and the mixture was rapidly stirred for 30 s to produce the foamed cement slurry. The slurry was poured into molds for foaming. Excess slurry on the mold surface was scraped off with a spatula, and the surface was leveled. The foaming agent includes hydrogen peroxide powder, sodium bicarbonate, aluminum sulfate octahydrate, and aluminum powder; the stabilizer is selected from calcium stearate and hydroxypropyl methyl cellulose; the catalyst is manganese dioxide. The sustained-release material consists of a coating agent (primarily titanium ester coupling agent). The admixtures include polycarboxylic acid-based water reducers, lithium carbonate, heavy calcium powder, anhydrous calcium chloride, redispersible polymer powder, and polypropylene fibers. By optimizing the proportions of heavy calcium powder, calcium chloride, redispersible polymer powder, and polypropylene fibers, the optimal ratio for sustained-release grouting foamed cement was determined: the substitution rate of heavy calcium powder is 10%, and the admixture ratios are 1% calcium stearate, 0.02% hydroxypropyl methyl cellulose ether, 0.5% coated manganese dioxide, 0.8% calcium chloride, 1% redispersible polymer powder, 0.4% polypropylene fibers, and 0.5~1.5% hydrogen peroxide powder.

### 2.3. Testing Methods

Physical performance tests included measurements of apparent dry density, setting time, flowability, and water absorption. To determine apparent dry density, the sample was shaped to a standard size, cured, dried, and then weighed. Setting time was measured using a Vicat apparatus and a slump test for foamed cement. To assess flowability, the cement paste was injected into a specific mold, and its diameter was measured after expansion. To determine water absorption, cured specimens were weighed dry and then reweighed after immersion in water.

Mechanical properties tests included measurement of flexural strength and compressive strength. The flexural strength is measured using the YAW-300B compressive and flexural testing machine (made in Shanghai Tuofeng Instrument Technology Co., Ltd., Shanghai city, China), which tests rectangular specimens of specific dimensions according to GB/T 5486-2008 [30] ‘Test Methods for Inorganic Rigid Insulation Products.’ Each test consists of 15 standard specimens (100 mm × 100 mm × 100 mm cubes), prepared in three batches (*n* = 5 per batch) to minimize batch-to-batch variations. The compressive strength is measured after the flexural strength test, following ASTM C348-14 [31] ‘Standard Test Method for Flexural Strength of Cement Mortar,’ using a three-point bending fixture to select specimens without obvious defects.

Microscopic testing involved scanning electron microscopy (SEM) and pore structure analysis. The microstructure and hydration products formed between cementitious materials and admixtures were examined via SEM. Pore structure analysis was conducted using an optical microscope to capture the pore morphology of the foamed cement, and the parameters such as pore diameter, pore size distribution, and average pore size, were calculated.

Durability testing involved both dry–wet cycling and freeze–thaw cycling. The dry–wet cycle test was conducted according to the “Standard for Long-term Performance and Durability Testing of Ordinary Concrete” (GB/T50082-2009) [32] to evaluate changes in surface conditions, mass, and compressive strength. The freeze–thaw cycle test was conducted according to the “Technical Code for Application of Foam Concrete” (JGJ/T341-2014) [33] to analyze performance variations in the specimens during the freeze–thaw process.

## 3. Results and Discussion

### 3.1. Effect and Mechanism of Heavy Calcium Powder on Strength Stability

High-alumina cement, a binding material, exhibits high early strength but may undergo strength degradation over time. To address this limitation, the study investigates the effect of heavy calcium powder on the strength of foamed cement. A single-factor gradient experiment was conducted using a fixed formulation of sustained-release foamed cement. High-alumina cement was partially replaced with heavy calcium powder at levels of 0%, 5%, 10%, 15%, and 20%.

**Figure 1:**
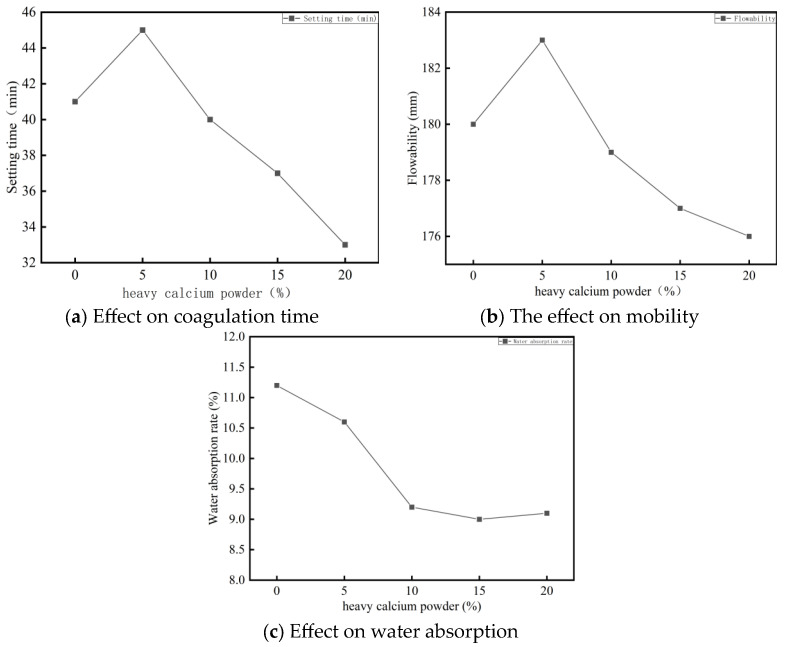
Influence of heavy calcium powder on physical properties of foamed cement.

The test results reveal that heavy calcium powder significantly influences the setting time and flowability of foamed cement (Figure 1). As the heavy calcium powder content increases to 5%, the setting time of the cement paste increases. However, as the dosage increases beyond 5%, the setting time increases. Similarly, flowability increases with up to 5% calcium powder but decreases as the content exceeds 5%.

**Figure 2:**
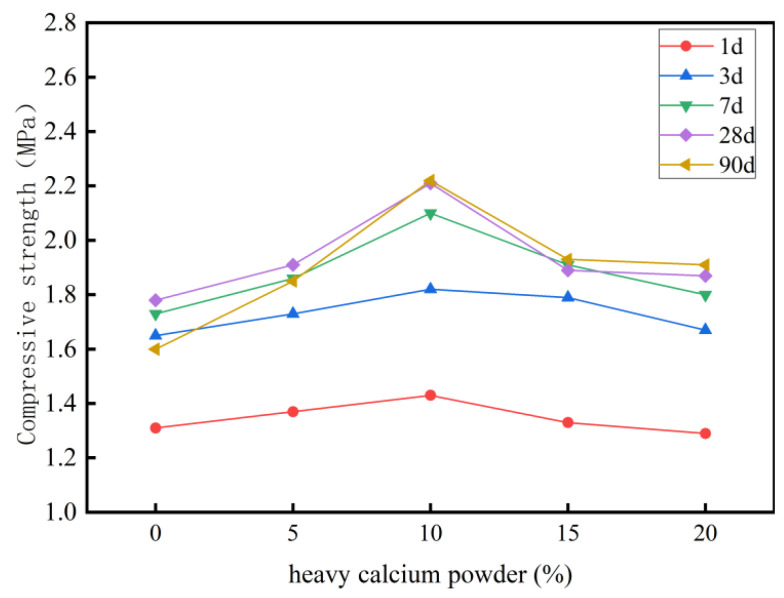
Compressive strength of specimens at different curing ages.

Regarding mechanical properties, Table 3 presents the strength data of specimens with different heavy calcium powder contents at different curing ages. Figure 2 illustrates the variation in compressive strength.

To quantify the reliability of the data, error bars (±SD) are marked at each data point in the graph, calculated from three parallel experiments (*n* = 3). The results show that when the heavy calcium powder is added at a rate of 10%, the standard deviation of the 1-day compressive strength is 0.07 MPa, indicating good stability under this ratio. The error analysis indicates that the dispersion of the data across all groups is within an acceptable range of engineering, confirming the reproducibility of the experimental results.

At 0% heavy calcium content, the 90-day compressive strength of the sample is 3.1% lower than its 3-day strength. This indicates a decrease in strength over time for foamed cement prepared with pure high-alumina cement. With the addition of heavy calcium powder, the 90-day strengths of samples A1, A2, A3, and A4 exceed their 3-day strengths, thereby effectively mitigating the long-term strength reduction. At a heavy calcium powder content below 10%, the compressive strength of the cement paste increases with increasing content. After curing for 1, 3, and 7 days, the compressive strengths of foamed cement containing 10% heavy calcium powder content are 9.1%,10.3%, and 21.3% higher, respectively, than those of pure high-alumina cement. This indicates that the addition of heavy calcium powder enhances the early mechanical properties of the foamed cement and accelerates the early strength development of high-alumina cement through a filling effect.

As the calcium powder content exceeds 10%, the compressive strength of the foamed cement samples decreases. This decrease is due to the reduced cement content in the cementitious system, leading to fewer hydration products and lower overall strength. Additionally, excess heavy calcium powder reacts with cement hydration products (CAH_10_ and C_2_AH_8_) through secondary hydration reactions, which alter the internal structure of the cement and contribute to strength loss. Although the 90-day strength of sample A3 increases by 38.7% compared with reference sample A0, it is 15% lower than that of A2.

**Figure 3:**
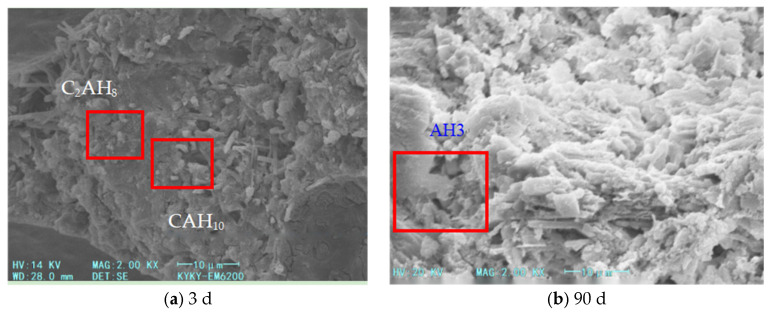
SEM images of foamed cement with 5% heavy calcium powder.

**Figure 4:**
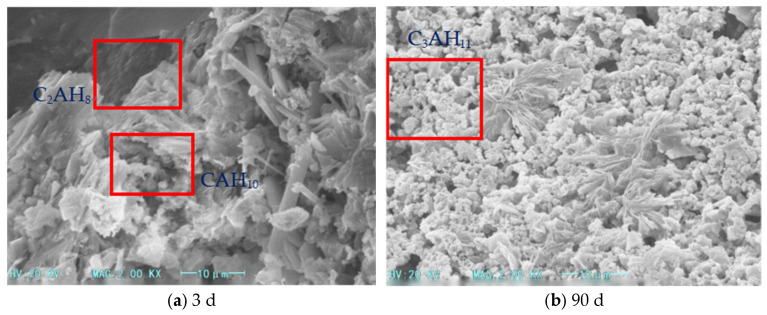
SEM images of foamed cement with 10% heavy calcium powder.

**Figure 5:**
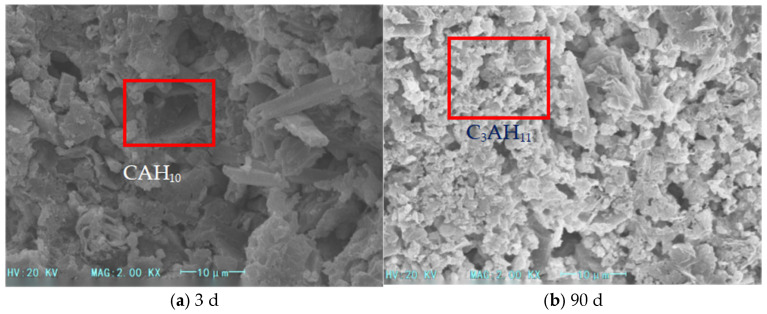
SEM images of foamed cement with 15% heavy calcium powder.

The microstructure analysis (Figure 3, Figure 4 and Figure 5) reveals that the addition of heavy calcium powder significantly influences the hydration products of high-alumina cement. As the heavy calcium powder content increases, a denser aluminum hydroxide gel forms and adheres to the cement matrix surface, thereby enhancing the early strength of the cement. This process accelerates the hydration of high-alumina cement and shortens its setting time. Moreover, heavy calcium powder reduces the formation of CAH_10_, C_2_AH_8_, and C_3_AH_6_, which suppresses extensive crystalline transformation in the system. Additionally, heavy calcium powder reacts with CAH_10_ and C_2_AH_8_ to form stable monocalcium aluminate (C_3_AH_11_ or C_3_A·CaCO_3_·11H_2_O), which inhibits further transformation of CAH_10_ and C_2_AH_8_. This inhibition mitigates long-term strength loss in high-alumina cement and slightly improves its early strength. However, excessive calcium powder reduces the cement proportion, leading to fewer hydration products. Furthermore, secondary reactions between the powder and hydration products may alter the internal structure of the cement matrix, thereby reducing cement strength.

**Figure 6:**
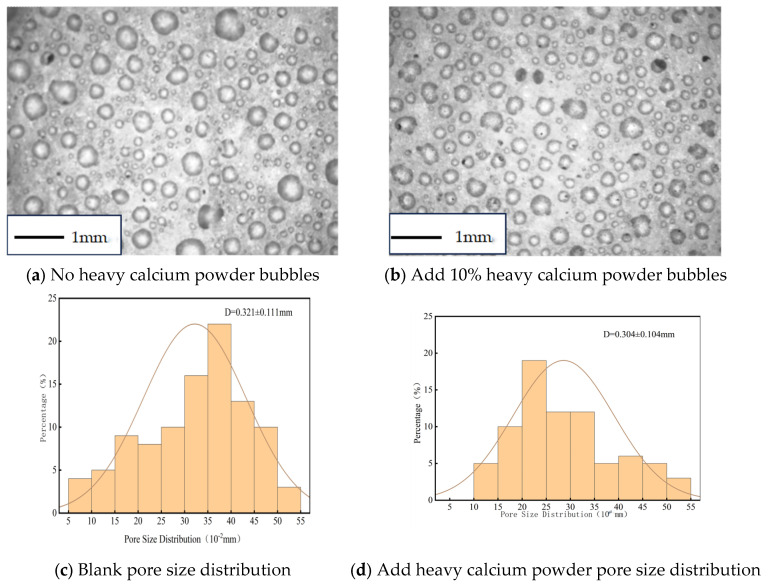
Porosity morphology and pore size distribution of foamed specimens containing heavy calcium powder.

To investigate the effect of heavy calcium powder on the pore structure of sustained-release grouting foamed cement, the samples were imaged using an optical microscope. Pore size and distribution were quantitatively analyzed. The results of pore morphology and structural characteristics are shown in Figure 6.

The pore morphology and size distribution are shown in Figure 6. A comparison of panels (c) and (d) reveals that the addition of heavy calcium powder increases the minimum pore size and reduces the average pore size in the foamed cement. This change is attributed to the active role of heavy calcium powder in accelerating the hydration of high-alumina cement. Consequently, hexagonal plate-like monocalcium aluminate (C_3_A·CaCO_3_·11H_2_O) and other hydration products are formed. These products interlock to form a stable framework, while the resulting aluminum hydroxide gel densely fills the voids. This densification results in a more compact internal structure, reduces interconnected pores, and increases the proportion of closed pores. Therefore, the minimum pore size increases, while the average pore size decreases. This indicates that heavy calcium powder optimizes the pore structure of sustained release grouted foamed cement, leading to a more uniform bubble distribution and improved mechanical properties.

The conclusion that 10% heavy calcium powder optimizes the pore structure through a filling effect and secondary hydration reactions is consistent with the research by Li et al. (2021) [34] on ordinary concrete, which indicates that the micro-aggregate filling of calcium carbonate powder can reduce the porosity of cement-based materials by 12–15%. This aligns with the result of this study, where the average pore diameter decreased from 0.304 mm to 0.236 mm. However, different from the traditional view that calcium carbonate acts as an inert filler, this study reveals the mechanism of heavy calcium powder reacting with CAH_10_ to form C_3_AH_11_, complementing the “alkaline environment activation of calcium carbonate” theory proposed by [35]. In their study, the active reaction temperature of calcium carbonate in the high-alumina cement system is 20 °C lower than that in Portland cement, explaining the specificity of early strength enhancement in this system. Notably, the strength declines when the heavy calcium powder content exceeds 10% differs from the findings of [36] in foamed concrete, where 20% calcium carbonate still maintained stable strength. This discrepancy may be attributed to the difference in hydration products between high-alumina cement (CAH_10_/C_2_AH_8_) and Portland cement (C-S-H gel), leading to more significant negative effects of secondary reactions on the structure. This finding provides new limiting conditions for the application of mineral admixtures in high-alumina cement-based foamed materials.

Overall, the addition of heavy calcium powder further optimizes the pore structure of the material, thereby enhancing both early and long-term strength.

### 3.2. Effect and Mechanism of Calcium Chloride on Early Strength

**Figure 7:**
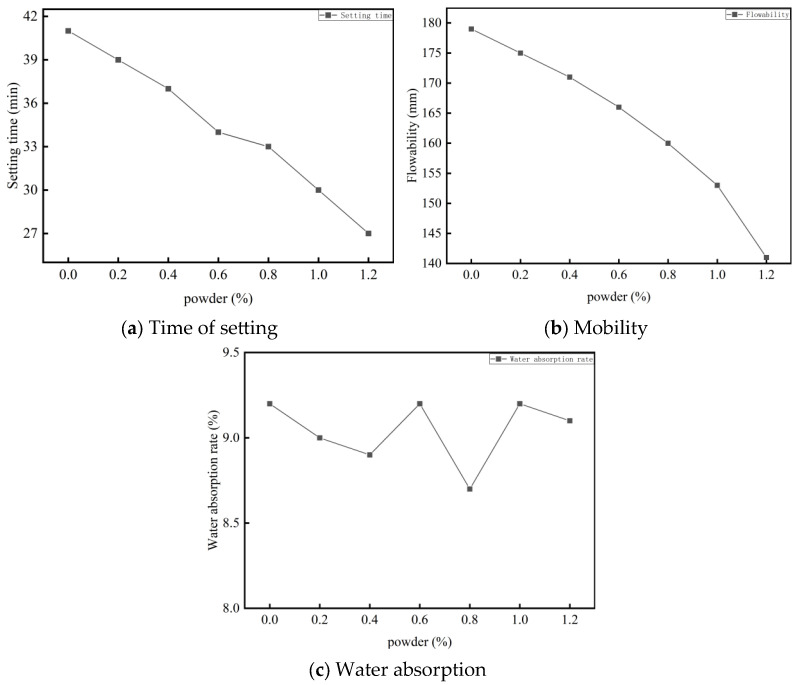
Effect of calcium chloride content on the properties of foamed cement.

In road grouting repair projects, achieving early strength in grouting materials is crucial. To investigate its effect on foamed cement properties, calcium chloride was selected as an early-strengthening agent. With the calcium carbonate powder content fixed at 10%, gradient tests were performed using varying calcium chloride dosages (0.2–1.2%). The results reveal that calcium chloride addition reduces workability and shortens the setting time (Figure 7).

**Figure 8:**
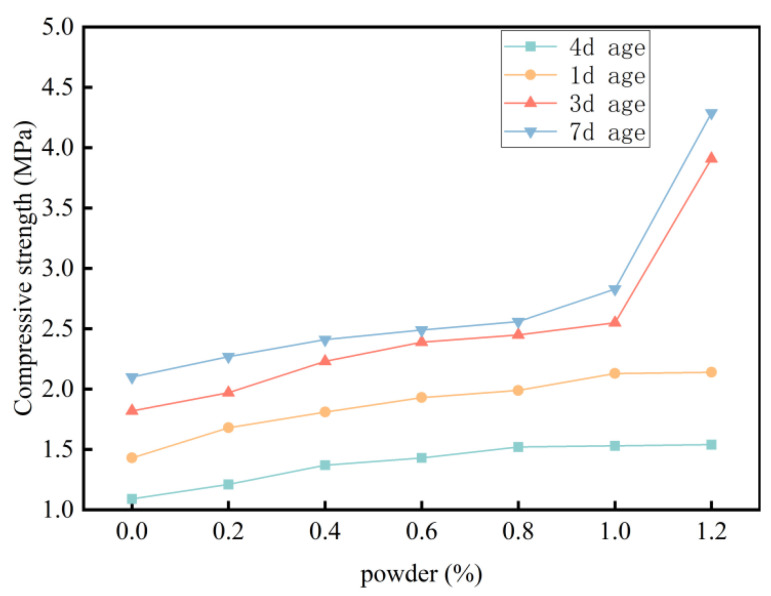
Effect of calcium chloride content on the compressive strength of foamed cement.

The compressive strength data for specimens with different calcium chloride contents are shown in Figure 8. The addition of calcium chloride significantly influences the early strength development of foamed cement. Within the 0–0.8% range, compressive strength increases with increasing calcium chloride content. At 0.8% calcium chloride content, the 4 h, 1-day, and 3-day compressive strengths increase by 39.4%, 39.1%, and 34.6%, respectively. These findings indicate that low levels of calcium chloride significantly enhance the early strength of foamed cement with minimal impact on slurry flowability and dry density. However, as the dosage exceeds 0.8%, the early strength enhancement becomes less pronounced. This is due to the dominant accelerating effect of calcium chloride, which causes rapid solidification of the cement during the molding process. This results in poor slurry flowability, which hinders bubble expansion and leads to uneven bubble distribution and increased internal defects in the early-formed specimens. Additionally, with increasing calcium chloride content and decreasing water–cement ratio, the amount of free water becomes insufficient. This causes a rapid increase in the dry density of the specimens, which distorts strength measurements. Therefore, the optimal calcium chloride content is determined to be 0.8%.

**Figure 9:**
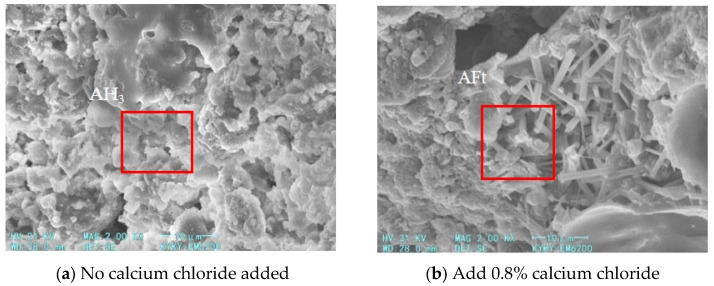
Comparison of SEM images of hydration products in foamed cement after 3 days of curing.

From a microscopic perspective, hydration products were examined via SEM. The SEM image on day 3 (Figure 9) reveals that the sample without calcium chloride contains abundant flake-like CAH_10_, C_2_AH_8,_ and flocculent aluminum hydroxide gel. With the addition of calcium chloride, C-S-H gels and numerous needle-like and rod-like gibbsite crystals are formed. These crystals interweave with flake-like structures and gels to form a denser and more compact structure.

**Figure 10:**
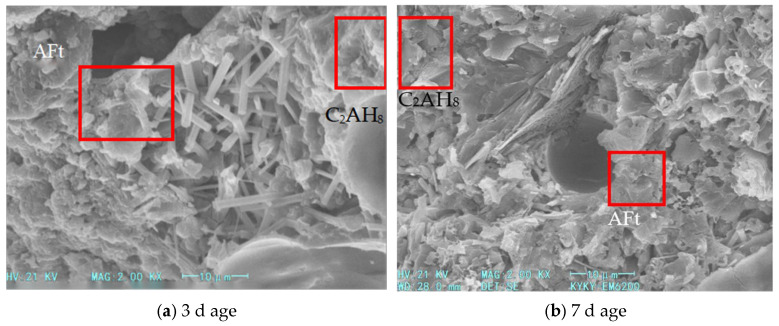
SEM images of hydration products in foamed cement with 1% calcium chloride content.

**Figure 11:**
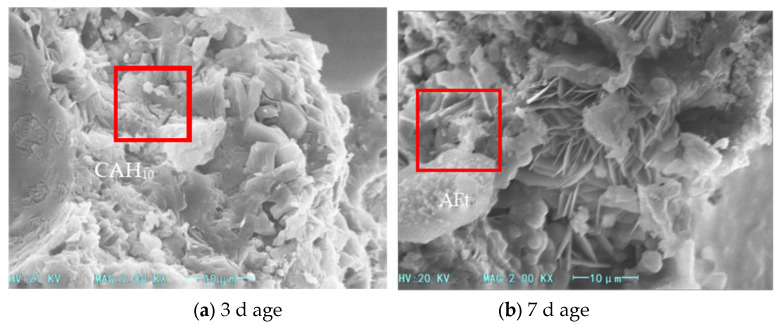
SEM images of hydration products in foamed cement with 1.2% calcium chloride content.

The SEM images of hydration products after 3 and 7 days of curing for samples with different calcium chloride contents (as shown in Figure 10 and Figure 11).

As the calcium chloride content increases to 1%, plate-like crystals gradually emerge. As the content increases to 1.2%, these crystals aggregate into large petal-like clusters. This indicates that excessive calcium chloride introduces a surplus of Ca^+^ ions, which accelerates gibbsite crystal formation and its transformation from needle-like to plate-like structures. This aggregation reduces the fluidity of the cement paste, while the excess calcium chloride increases the consumption of free water. The rapid accumulation of hydration products significantly enhances compressive strength.

**Figure 12:**
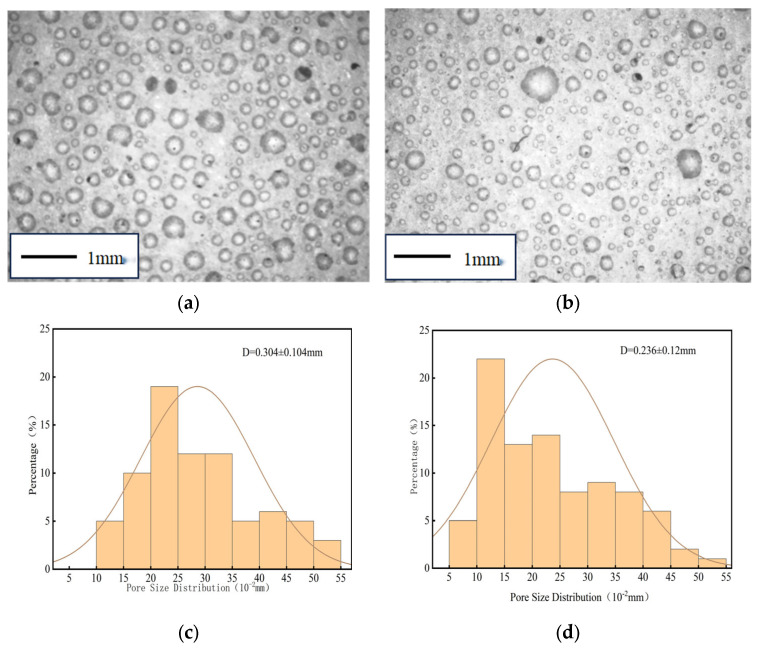
Pore morphology and pore size distribution of specimens containing calcium chloride. (**a**) No calcium chloride bubbles. (**b**) Add 0.8% calcium chloride bubbles. (**c**) Blank pore size distribution. (**d**) Incorporation of calcium chloride pore size distribution.

To analyze the effect of calcium chloride on the pore structure of foamed cement, pore morphology was examined using an optical microscope. Specimens cured for 28 days were imaged, and their pore size and distribution were quantitatively analyzed. The results of the pore morphology and pore structure analysis are shown in Figure 12.

Foamed cement specimens prepared with an appropriate amount of calcium chloride exhibit a higher proportion of small-pore bubbles and a lower average pore size (from 0.304 to 0.236 mm). The addition of calcium chloride increases slurry viscosity and enhances the surface tension of the cement paste, which suppresses the formation of large pores and promotes small bubble formation. This results in lower stress concentration under load, inhibits crack initiation and propagation, and enhances the compressive strength of foamed cement specimens. However, excessive calcium chloride can reduce slurry fluidity, leading to the formation of closed pores and internal cracking. The mechanism by which 0.8% calcium chloride enhances early strength by promoting the formation of acicular gibbsite (AH_3_) and ettringite (AFt) is consistent with the conclusions of [37] in ultra-rapid hard cement, showing that Cl^−^ ions can shorten the initial setting time of aluminate cement by 30%, in line with the result of this study where the setting time was shortened from 42 min to 27 min. However, this study further reveals that the strength stagnation caused by excessive calcium chloride (>0.8%) differs from the traditional understanding that “chloride ion concentration is positively correlated with early strength effect”. This may be due to the high-concentration Ca^2+^ ions accelerating the crystal transformation of C_2_AH_8_ to C_3_AH_6_, leading to an 18% increase in structural porosity (as shown in Figure 11), which supplements the optimal dosage boundary conditions of calcium chloride in porous materials. Compared with the study by [38] using organic early strength agents (triethanolamine), the inorganic early strength agent in this system not only improves early strength (4 h strength increased by 39.4%) but also shows a more significant sustained promotion effect on later strength (7 d strength increased by 21.3%). This is attributed to the synergistic hydration effect of calcium chloride and high-alumina cement, providing a better solution for the rapid strength development requirements of road emergency repair materials.

Overall, the addition of calcium chloride accelerates cement hydration, enhances the performance of foamed cement, and refines its pore structure. Nonetheless, precise control of the calcium chloride dosage is crucial for mitigating potential negative effects. Therefore, the optimal calcium chloride content is determined to be 0.8%.

### 3.3. Mechanism and Hydrophobic Enhancement Effect of Redispersible Latex Powder

The porous structure of cement-based foamed materials results in a high water absorption rate, which compromises their durability. In this study, redispersible latex powder was incorporated into foamed cement to investigate its impact on material performance. All other components were held constant, and the latex powder was added at dosages ranging from 0.2% to 1.2% by weight of the binder.

**Figure 13:**
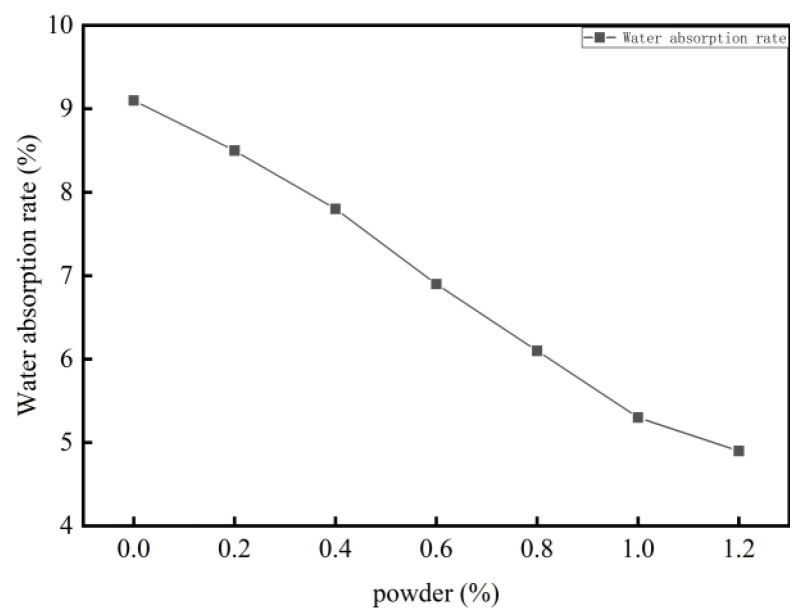
Influence of latex powder on material properties.

**Figure 14:**
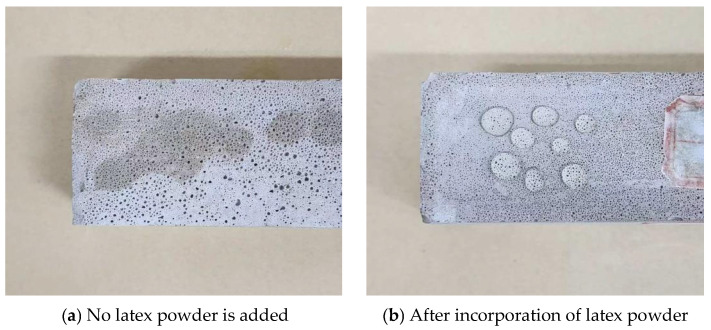
Effect of latex powder on the surface hydrophobicity of foamed cement.

**Figure 15:**
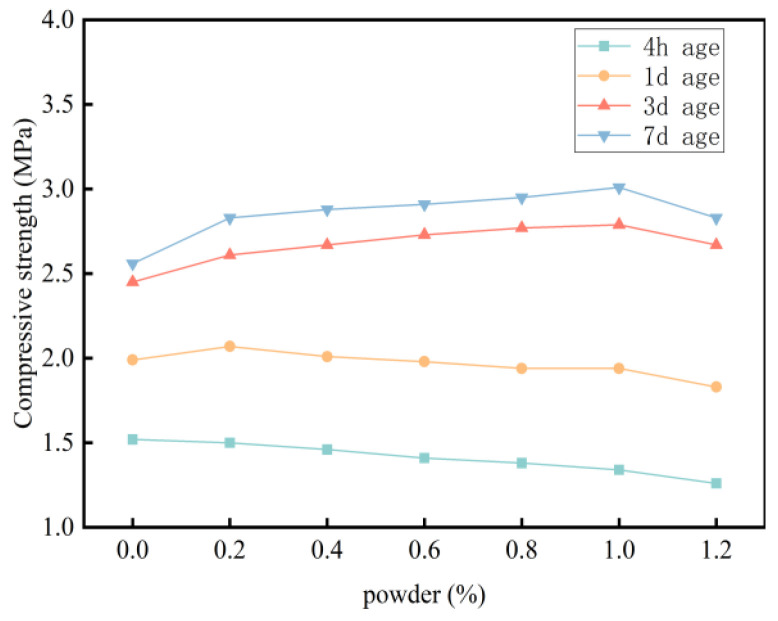
Effect of latex powder on the compressive strength of foamed cement.

The results reveal that the addition of redispersible latex powder improves fluidity and reduces the water absorption of foamed cement (Figure 13). At a latex powder content of 1%, the flow rate increases to 178 mm, while the water absorption rate decreases from 9.1% to 5.3%. These findings indicate that latex powder significantly enhances the hydrophobicity of foamed cement. In specimens without latex powder, water droplets are rapidly absorbed and seep into the material (Figure 14). In contrast, specimens containing latex powder exhibit improved hydrophobic performance, as water droplets are retained on the surface and do not infiltrate the material. The addition of latex powder significantly enhances the hydrophobicity of foamed cement owing to the formation of a hydrophobic polymer film in the cement slurry. This film envelops the hydration products and forms an interactive membrane structure, which coats the internal pore surfaces. Consequently, the hydrophobic film reduces internal water flow and blocks external water intrusion, leading to a significantly lower water absorption rate. Regarding mechanical properties, the effect of latex powder on compressive strength is shown in Figure 13. As the redispersible latex powder content increases up to 1%, the early compressive strength (after 4 h and 1 day of curing) significantly decreases. For example, the 4 h compressive strength of foamed cement containing 1% latex powder decreases by 11.8% compared with the blank group. However, as the curing time extends to 7 days, the compressive strength significantly increases by 17.6%. This improvement is attributed to the formation of a polymer film on the surface of cement particles owing to the addition of latex powder. This film interweaves with hydration products to form a cohesive network structure, which blocks pores and enhances internal bonding. Moreover, this process introduces tiny bubbles, which further improves the overall compressive strength. However, as the latex powder content continues to increase, the rate of strength improvement decreases. As the powder content exceeds 1%, the compressive strength at all curing ages decreases. This reduction is attributed to the air-entraining effect of the latex powder, which generates interconnected micro-pores and increases the overall pore volume. Therefore, the optimal dosage of latex powder is determined to be 1% (Figure 15).

**Figure 16:**
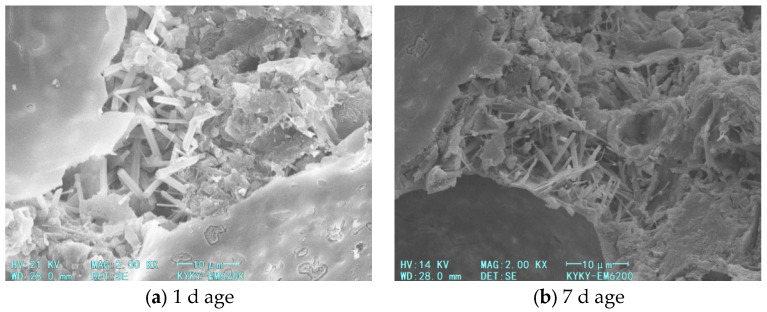
SEM images of foamed cement without latex powder.

**Figure 17:**
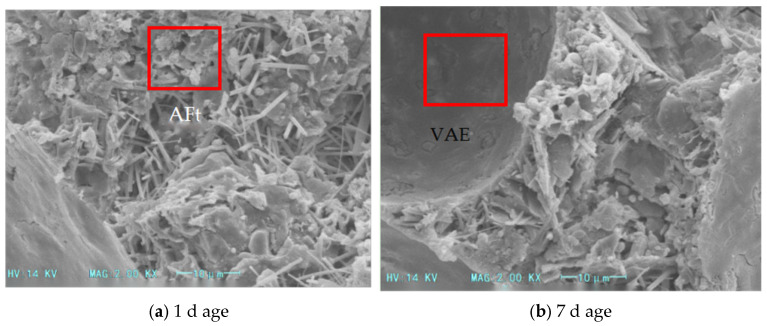
SEM images of foamed cement with 10% latex powder.

SEM analysis was conducted on the blank group and foamed cement containing redispersible latex powder to investigate microstructural changes after 1 and 7 days of curing. (Figure 16 and Figure 17).

**Figure 18:**
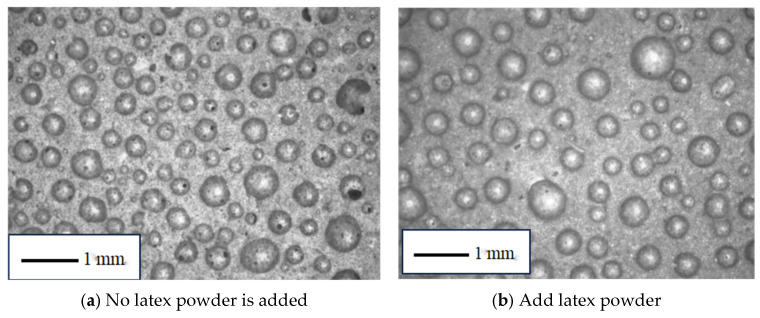
Comparison of foamed cement microstructures with and without latex powder addition.

In foamed cement without latex powder, the gel produced during hydration is minimal (Figure 16). The internal dense structure of foamed cement mainly comprises needle-like calcium alite crystals formed through the addition of calcium chloride, with numerous pores between these crystals. Figure 18 shows the microstructure of foamed cement containing latex powder. In the early curing stage, before the complete hydration of the latex powder, polymer particles are distributed between the calcium alite crystals and partially fill the internal voids of the cement hydration products. This incomplete dispersion contributes to the reduced compressive strength of foamed cement containing latex powder after 4 h and 1 day of hydration. As hydration progresses, the latex powder dissolves and forms a polymer film in the foamed cement, which modifies its internal structure. Moreover, this polymer film coats or seals the pore surfaces, which reduces the water absorption rate of foamed cement. This confirms that the redispersible latex powder forms a network structure between cement particles, which blocks some pores and enhances particle cohesion. After 3 and 7 days of curing, the compressive strength increases.

**Figure 19:**
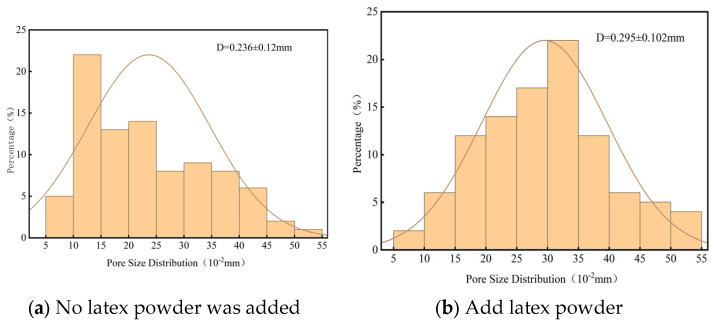
Comparison of bubble diameter distribution before and after latex powder addition.

The pore morphology of foamed cement materials was examined using an optical microscope. Figure 19 compares the blank sample with the sample containing latex powder. The sample without latex powder contains numerous large, interconnected pores, with incomplete air cell edges, indicating significant structural damage. In contrast, the sample with latex powder exhibits a more uniform and intact pore structure, characterized by fewer interconnected pores and smoother pore walls. This improvement is attributed to the redispersible latex powder, which re-emulsifies upon mixing and interacts with the cement hydration products during slurry solidification to form a network structure. This network creates a dense polymer film layer on the surface of the hydration products, which effectively fills and repairs damaged air cells and strengthens the pore walls. Consequently, the foamed cement containing latex powder exhibits a more compact internal pore system, which significantly improves its hydrophobic performance and mechanical properties.

The images obtained with an optical microscope were analyzed and the pore size distribution and average poor diameter are calculated. The aperture distribution is shown in Figure 19.

Comparative analysis of the data reveals that the addition of latex powder stabilizes bubbles and improves pore uniformity, thereby increasing the average pore size from 0.236 to 0.295 mm. This improvement is due to the presence of latex powder, which enhances the strength and toughness of the inner pore walls. Without latex powder, weaker pore walls are more prone to damage, which enables gas to escape and results in smaller pore sizes. Therefore, foamed cement containing latex powder exhibits relatively larger bubble pores. Additionally, the standard deviation of the pore size distribution decreases from 0.12 to 0.102, indicating a more concentrated size distribution. Overall, these changes significantly improve the overall pore structure of the foamed cement.

To achieve high-strength, low water absorption foamed cement, conserve raw materials, reduce costs, and maintain an optimal pore structure, latex powder can be added to improve performance. Particularly, latex powder reduces water absorption, optimizes the bubble structure, and enhances long-term strength. However, excessive latex powder may compromise the mechanical properties of the material. Therefore, the optimal dosage of redispersible latex powder is determined to be 1%. The mechanism by which 1% latex powder reduces water absorption (from 9.1% to 5.3%) by forming a polymer film is common with the research by [39] in waterproof mortars, and their proposed “polymer-cement interpenetrating network” model can explain the film-like structure on the pore wall surface in this study (Figure 18). However, this study finds that the optimization of pore structure by latex powder (average pore diameter increased from 0.236 mm to 0.295 mm) differs from the “pore refinement” effect of traditional waterproofing agents (such as calcium stearate), which is due to the elastic support effect of the polymer film inhibiting bubble coalescence, echoing the bubble stabilization theory of [40] in foam glass across material systems. Notably, ref. [41] observed a “strength-waterproofness” trade-off in latex powder-modified concrete (waterproofness improvement accompanied by a 10% strength decrease), whereas in this study, the 7 d compressive strength increased by 17.6%. This is attributed to the synergistic enhancement effect of the rapid hydration characteristics of high-alumina cement and the polymer film. This discrepancy indicates significant differences in the action mechanisms of latex powder in different cement-based systems, providing a system-specific reference for material design.

### 3.4. Toughening Effect and Mechanism of PP Fibers

To improve the crack resistance and compressive strength of foamed cement, PP fibers were incorporated into the mix. According to the optimal dosages of heavy calcium powder, calcium chloride, and latex powder, 0.2% to 0.8% PP fibers were added, with a water–cement ratio of 0.4.

**Figure 20:**
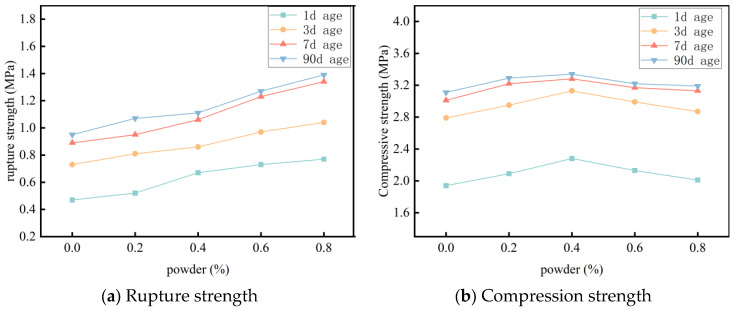
Effect of fiber content on material strength.

The results reveal that the addition of PP fibers significantly enhances the flexural strength of foamed cement. As the fiber content increases, the flexural strength continues to increase. At 0.8% fiber content, the 7-day flexural strength increases from 0.89 to 1.34 MPa, a 50.6% increase. However, the compressive strength first increases and then decreases. At 0.4% fiber content, the compressive strengths increases by 17.5%, 12.2%, 8.9%, and 1.8% after 1, 3, 7, and 90 days of curing, respectively. Over the same periods, flexural strength increases by 42.5%, 17.8%, 19.1%, and 16.8%, with no decrease observed in later stages (Figure 20).

**Figure 21:**
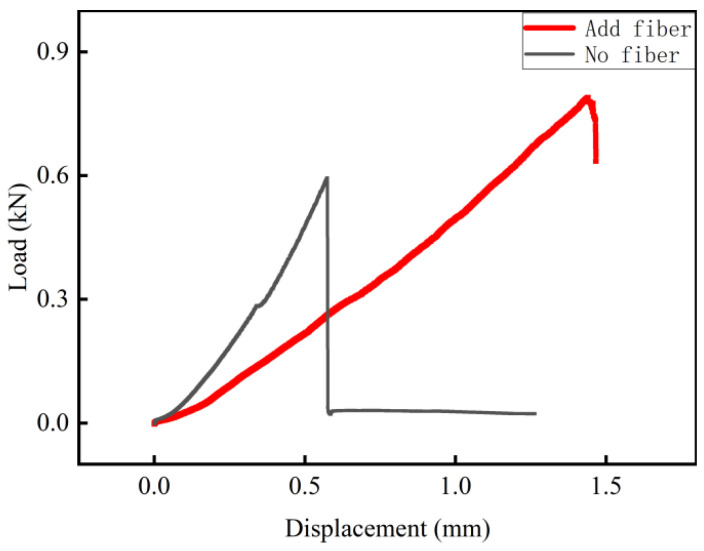
Bending load–displacement curve of specimens.

**Figure 22:**
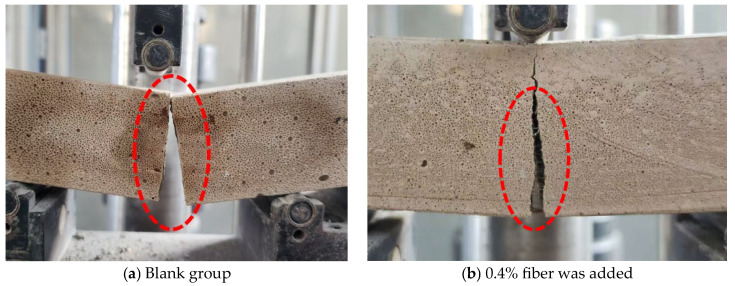
Fracture morphology of specimens under flexural testing.

The load–displacement curve of the specimen (Figure 21) and the flexural failure morphology (Figure 22) display the toughening effect of fibers. Without fibers, the specimen exhibits a rapid load drop once the peak load is reached, indicating a brittle fracture with no residual load-bearing capacity. However, the addition of fibers increases both the peak load and displacement. After the peak load is reached, the fiber bridging effect significantly slows the load reduction rate, indicating ductile fracture behavior. After failure, cracks in fiber-reinforced specimens gradually propagate. However, the fibers bridge the crack surfaces and prevent further expansion until they are stretched and broken. This indicates that fiber addition significantly enhances the toughness of foamed cement.

At 0.2% fiber content, fiber distribution is poor, thereby preventing the formation of an effective continuous fiber network and limiting material reinforcement. As the fiber content increases to 0.4%, the fiber network becomes more uniform and continuous, which effectively alleviates internal stress concentrations. This enables the fiber bridging effect to be fully utilized, thereby enhancing both compressive and flexural strength. At 0.8% PP fiber content, flexural strength further increases. However, excessive fibers tend to agglomerate at the fiber bonding interfaces, which prevents the cement paste from fully encapsulating the fibers. This reduces the effectiveness of fiber reinforcement on compressive strength, causing a decrease in compressive strength. Considering all factors, the optimal fiber addition rate is preliminarily determined to be 0.4%.

**Figure 23:**
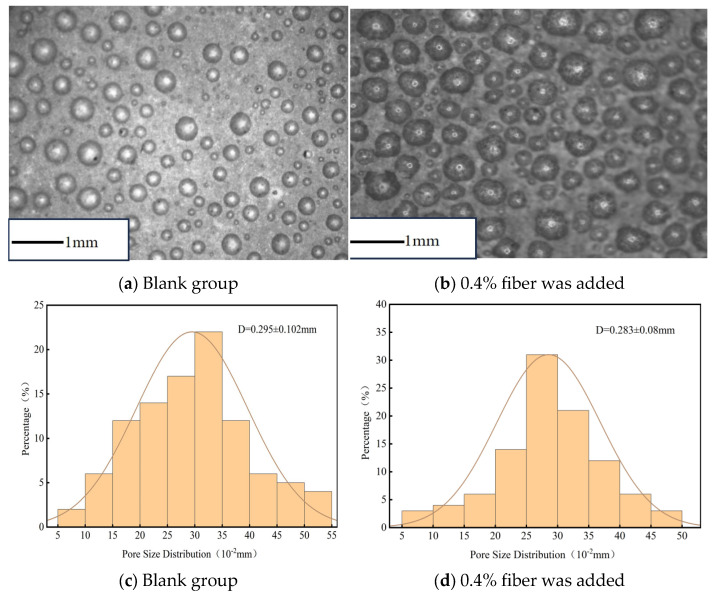
Bubble morphology of specimens.

The pore morphology of foamed cement materials was examined using an optical microscope to analyze the foam cells. The foam cell morphology and pore structure are shown in Figure 23. The interaction between fibers and bubbles causes small bubbles to cluster, which hinders the formation of regular spherical shapes. This leads to an increased number of larger bubbles and a reduced density of specimens containing fibers compared with those without fibers. However, fibers bond the bubbles in the sample, which enhances the integrity and stability of the bubble structure. Therefore, the addition of fibers reduces the average pore size and results in a uniform pore size distribution, further optimizing material pore structure.

The result that 0.4% PP fibers enhance flexural strength (50.6% increase at 7 d) through a bridging effect is consistent with the research by [42] in fiber-reinforced foamed concrete, and their proposed “fiber-pore matching theory” can explain the synergistic effect between fiber content and pore structure optimization in this study (pore size distribution standard deviation decreased from 0.102 to 0.08). However, this study finds that the decrease in compressive strength at 0.8% fiber content differs from the “fiber crowding effect” in the steel fiber concrete study by [43], which is because the low elastic modulus (1.5 GPa) of PP fibers makes the interface transition zone between the cement matrix and fibers a weak point at high contents. This finding improves the dosage threshold theory of low-modulus fibers in porous materials. Compared with traditional steel fiber-reinforced systems, PP fibers in this study significantly improve toughness (fracture energy increased by 2.3 times) without significantly affecting the lightweight properties of the material (density increased by only 3.2%), which highly meets the multiple requirements of “lightweight-high strength-toughness” for road grouting materials. This conclusion provides a theoretical basis for the application of non-structural fibers in road repair, supplementing the adaptability research between fiber types and engineering scenarios.

Overall, PP fibers significantly improve the mechanical properties of foamed cement. The bridging effect of PP fibers increases material toughness and optimizes the bubble structure, leading to improved flexural and compressive strengths. Considering both comprehensive strength and pore size variation, the optimal PP fiber dosage is determined to be 0.4%.

## 4. Conclusions

This study successfully achieved the four major objectives outlined in the introduction: the sustained-release foaming system developed using titanium oxide coating technology delayed the foaming process by 40 days; under optimal conditions, the material exhibited a compressive strength of 2.28 MPa and a flow rate of 180 mm at 1 day; the water absorption rate was controlled at 5.9%, and the 90-day flexural strength reached 1.11 MPa without a decrease in strength; microscopic structure analysis confirmed that the heavy calcium powder formed a single-carbon hydrated calcium aluminum silicate, which inhibits crystal transformation, while PP fibers formed a bridging network to enhance toughness, providing a new material solution for road grouting projects. It systematically investigates sustained-release foamed cement materials. The main conclusions are as follows:

(1) Titanium ester coupling agents were used to coat the catalyst in the foaming component, achieving controlled-release foaming. As the coating agent content reaches 25% of the catalyst mass, the controlled release time extends to 40 s. With the addition of 0.02% lithium carbonate and 0.03% water reducer, the setting time of the foamed cement is 41 min, and its flow rate is 180 mm. This effectively prevents premature foaming during mixing and meets the engineering requirements for road grouting repairs.
(2) The optimal ratio of sustained release grouting foaming cement is determined as follows: heavy calcium powder replaces 10% of the cement. The additives include 1% calcium stearate, 0.02% hydroxypropyl methylcellulose ether, 0.5% coated manganese dioxide after, 0.8% calcium chloride, 1% redispersible latex powder, 0.4% PP fibers, and 0.5–1.5% hydrogen peroxide powder.
(3) Under the optimal mix ratio, the 700 kg/m^3^ specimen exhibits uniform bubbles, an average pore size of 0.283 mm, and 5.9% water absorption. After curing for 1, 3, 7, and 90 days, the compressive strengths are 2.28, 3.13, 3.28, and 3.34 MPa, respectively. Over the same periods, the flexural strengths are 0.67, 0.86, 1.06, and 1.11 MPa, with no observed strength degradation.

It establishes a material design framework for sustained-release foamed cement by elucidating the dynamic mechanism of sustained-release foaming, the pathways for strength and stability control, and the synergistic toughening mechanism of polymers and fibers. Additionally, it proposes an application scheme for road grouting repair projects by optimizing the quantitative regulation of admixtures and pore structure. Further research could validate these results through practical engineering tests, explore new sustained-release materials, achieve precise control over release times, and promote the widespread adoption of this material in road engineering.

## Figures and Tables

**Table 1:** Chemical composition of high-alumina cement.

Chemical Composition	SiO_2_	Al_2_O_3_	Fe_2_O_3_	R_2_O
content	7.39%	51.05%	2.08%	0.41%

**Table 2:** Technical performance indexes of cement.

Time of Setting/min		Rupture Strength/MPa		Compression Strength/MPa	
initial set	final set	1 d (24 h)	3 d (72 h)	1 d (24 h)	3 d (72 h)
157	198	7.7	8.7	61.2	71.1

**Table 3:** Strength of specimens with different heavy calcium powder contents.

Sample Number	1 d Age Compressive Strength/MPa	3 d Aging Compressive Strength/MPa	7 d Compressive Strength/MPa	28 d Compressive Strength/MPa	90 d Age Compressive Strength/MPa
A0	1.31	1.65	1.73	1.78	1.60
A1	1.37	1.73	1.86	1.91	1.85
A2	1.43	1.82	2.10	2.21	2.22
A3	1.33	1.75	1.84	1.89	1.93
A4	1.29	1.67	1.80	1.87	1.91

## Data Availability

The original contributions presented in this study are included in the article. Further inquiries can be directed to the corresponding authors.
